# Evaluation of the Quantitative and Qualitative Alterations in the Fatty Acid Contents of the Sebum of Patients with Inflammatory Acne during Treatment with Systemic Lymecycline and/or Oral Fatty Acid Supplementation

**DOI:** 10.1155/2013/120475

**Published:** 2013-09-26

**Authors:** Adilson Costa, Aline Siqueira Talarico, Carla de Oliveira Parra Duarte, Caroline Silva Pereira, Ellem Tatiani de Souza Weimann, Lissa Sabino de Matos, Livia Carolina Della Coletta, Maria Carolina Fidelis, Thaísa Saddi Tannous, Cidia Vasconcellos

**Affiliations:** ^1^Service of Dermatology of the Pontifical Catholic University of Campinas, Campinas, SP, Brazil; ^2^KOLderma Clinical Trials Institute, Campinas, SP, Brazil; ^3^Department of Dermatology of the University of Sao Paulo, Sao Paulo, SP, Brazil

## Abstract

*Background*. Acne is a dermatosis that involves an altered sebum pattern. *Objectives*. (1) To evaluate if a treatment based on antibiotics (lymecycline) can alter fatty acids contents of the sebum of patients with acne; (2) to evaluate if oral supplementation of fatty acids can interfere with fatty acids contents of the sebum of patients with acne; (3) to evaluate if there is any interaction in fatty acids contents of the sebum of patients with acne when they use both antibiotics and oral supplementation of fatty acids. *Methods*. Forty-five male volunteers with inflammatory acne vulgaris were treated with 300 mg of lymecycline per day, with 540 mg of *γ*-linolenic acid, 1,200 mg of linoleic acid, and 510 mg of oleic acid per day, or with both regimens for 90 days. Every 30 days, a sample of sebum from the forehead was collected for fatty acids' chromatographic analysis. *Results*. Twelve fatty acids studied exhibited some kind of pattern changes during the study: C12:0, C14:0, C15:0, C16:1, C18:0, C18:1n9c+C18:1n9t, C18:2n6t, C18:3n6, C18:3n3, C20:1, C22:0, and C24:0. *Conclusions*. The daily administration of lymecycline and/or specific fatty acids may slightly influence some fatty acids levels present in the sebum of patients with inflammatory acne vulgaris.

## 1. Introduction

Acne is a chronic dermatosis that affects the pilosebaceous follicles. The physiopathogenesis of this condition involves periglandular dermal inflammation mechanisms, sebum hyperproduction, follicular hyperkeratosis, an increase of colonization of *Propionibacterium acnes *(*P. acnes*), and hormones [1–3]. We have observed that many of these pathogenic mechanisms are governed by a bio-immuno-molecular phenomenon that serves as the basis for research on and the future development of possible individual treatments for this dermatosis [4].

Fatty acids (FAs) constitute an essential part of corporeal lipids, especially FAs with chains composed of 12 to 24 carbons and 0 to 6 double bonds [5]. Essential fatty acids (EFAs) are FAs that the body is not capable of producing, such as linoleic acid (LA, 18:2n-6) and *α*-linolenic acid (ALA, 18:3n-3), which cannot be produced due to lack of Δ12- and Δ15-desaturases [5]. In the human body, EFAs are primarily found in phospholipids and in the triglycerides (TGs) which participate in sebum (SB) formation [6].

SB of patients with AV is different from those without this condition. One of the main characteristics is the lower concentration of LA, and this low AL concentration facilitates follicular hyperkeratosis [7–9], SB hyperproduction [3, 10, 11], and periglandular dermal inflammation [12]. LA is present in acyl-glycosyl ceramides; hence, it participates in the maintenance of the skin barrier [13]. If LA is absent from the SB, the integrity of the skin barrier is compromised, and the free fatty acids (FFAs) resulting from the hydrolysis of TGs by the lipases of *P. acnes* [14] act on the infundibular epithelium, causing hyperkeratosis and inflammation [3, 10, 11].

Antibiotics can alter the composition of the SB of patients with AV; tetracycline antibiotics are the most effective [15]. Lymecycline (LM; 150 mg–300 mg/day) is a semisynthetic derivative antibiotic of the tetracycline group [16, 17]. There are no reports in the literature about the influence of lymecycline on SB.

An example of the systemic use of EFAs to treat skin diseases is the treatment of atopic dermatitis, a skin condition that results in the disruption of the skin barrier [18, 19]. For individuals with AV, there are reports of clinical benefits with the use of EFAs [20–22], including the possibility of reducing the size of the sebaceous glands (SGs) [10]. There are no reports in the literature about the influence of EFAs on the FA composition of SB, nor is there any indication that EFAs can be used for the treatment of acne [23].

Given that the LM is a modern antibiotic used for the treatment of acne [16, 17], we conducted a study to evaluate the possible clinical improvements of this dermatosis after treatment with this antibiotic and how these improvements are related to the variation of the FA composition of SB. In addition, because SB is rich in FAs [24–26] and because past studies have shown that the levels of some FAs are altered in the SB of patients with AV [7–10, 12], we added a daily supplement composed of FAs present in SB to determine whether these FAs could individually interfere with and/or maximize the possible alterations caused by LM in these patients. 

## 2. Methods

This study was approved by the Ethics Committees on Clinical Research of two universities and complied with all rules laid down by the Declaration of Helsinki. In this single-center, randomized, single-blind, parallel, and phase IV study, forty-five male volunteers with inflammatory AV (either papulopustular or cystic) were recruited from the Service of Dermatology of the Pontifitial Catholic University of Campinas (Campinas/SP, Brazil). Patients were between 12 and 40 years of age and had phototype I to VI (Fitzpatrick phototype scale). 

These volunteers were divided (by previous statistical randomization using the method of numerical drawing) into three treatment groups of 15 individuals each and were treated with the following regimens for 90 consecutive days: Group 1: 300 mg/day of LM (Tetralysal, Galderma do Brasil Ltda., Ortolândia/SP, Brazil); Group 2: 540 mg of *γ*-linolenic acid, 1,200 mg of linoleic acid, and 510 mg of oleic acid per day (Tiliv L, borage seed oil (*Borago officinalis*), Arese Pharma Ltda., Valinhos/SP, Brazil); and Group 3: the combination of the treatments used for Groups 1 and 2. The products were ingested with water at lunchtime. This study was conducted between February and October 2011.

The volunteers analyzed on the first study day (D0) returned for monthly consultations for 90 days (D90) to provide samples of facial SB (for chromatographic analysis), to receive treatment products (except on D90), and to allow us to assess therapeutic adherence and side effects. Over all visits, the volunteers were instructed to refrain from washing their face for 12 hours prior to the clinical evaluation. They also remained in a room with temperature set at 21°C for 30 minutes prior to the collection of SB.

### 2.1. Obtaining SB Samples

SB samples were obtained from a 32 cm² area of the forehead through multiple swabbings with sterile cotton swabs intended for commercial hygienic use. The cotton balls were removed from the plastic shafts and soaked with 4 drops of n-hexane. These cotton balls were placed in 2 mL vials and stored for transportation at a temperature of 4°C. 

### 2.2. Preparation of SB Samples

The cotton balls were placed in a sterile Erlenmeyer flask, to which were added 10 mL of methanolic KOH at 0.5 N and 8 glass beads. The flask was placed in a condenser that, after the heating-cooling-ebullition cycles were complete, was washed with 15 mL of sterilizing solution (3.8 g of ammonium chloride 99.5%, 115 mL of methanol, and 6 mL of concentrated sulfuric acid); another heating-ebullition cycle was performed. After 10 minutes, the Erlenmeyer flask was cooled with ice for 10 minutes. This condensed liquid was placed in 10 mL of n-hexane and was then shaken for 90 seconds. Finally, 2 mL was used for the chromatographic analysis. 

### 2.3. Chromatographic Analysis Method

**Figure 1:**
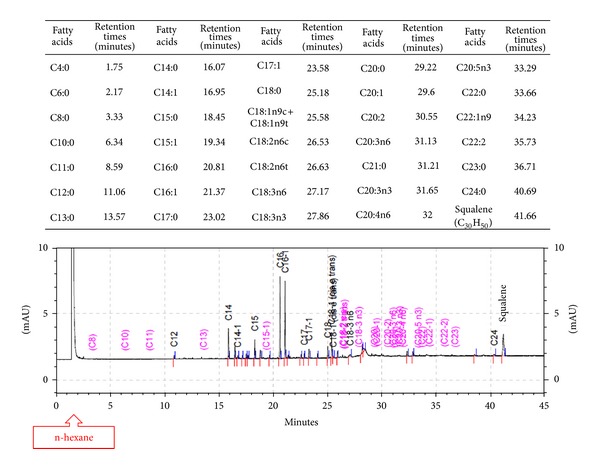
Evaluated fatty acids, their respective retention times, and example of chromatographic result graphic.

Gas chromatography was performed with a flame ionization detector (GC 7890 A, Agilent Technologies Brasil Ltda., Barueri/SP, Brazil) (GC-DIC) and an HP-Innowax polyethylene glycol column (length: 30 m; internal diameter: 0.25 mm; depth of stationary phase: 0.25 *μ*m) that was calibrated for analysis using polyethylene glycol at a dilution of 1 : 20. AFA standard was used (47885-U/602-004-00-3, Supelco 37 Component FAME Mix, Sigma-Aldrich Brasil Ltda., Sao Paulo/SP, Brazil), and a separate standard was used for squalene (S3626/111-02-4, Squalene, Sigma-Aldrich Brasil Ltda., Sao Paulo/SP, Brazil). The FAs analyzed and their respective retention times (RT) are presented in Figure 1.

After 45 minutes, the amount of each of the FA was obtained in milli-absorbance units (mAU) (Figure 1), and these values were converted into percentages in the sample. It should be noted that the first peak was for the solvent (n-hexane).

### 2.4. Statistical Methods

A significance level of 5% was used. The aim of this study was to evaluate the two different factors used in this study, groups and visits, and the differences were tested by two-way analysis of variance (ANOVA) with repeated measures. The Huynh-Feldt correction was used to determine any differences in the percentages of the various FAs in the SB.

 For the differences that were statistically significant, Bonferroni's correction for multiple comparisons was used to identify the circumstance(s) under which such difference(s) occurred. The data were analyzed with STATA version 11.0, SPSS version 17.0, and SAS version 8.0. 

## 3. Results

Five volunteers (11.1%) did not complete the study for personal reasons. No side effects were observed in any of the groups. The characteristics of the volunteers who completed the study were as follows: average age = 18.3 years; AV grade II = 82.5% (*n* = 33) and III = 17.5% (*n* = 7); phototype I = 22.5% (*n* = 9), II = 27.5% (*n* = 11), and III = 50% (*n* = 20); and the average duration of AV = 5.8 years. For all groups, all volunteers ingested the complete dosage of the products throughout the study. The groups did not differ significantly with respect to age (*P* = 0.626), acne grade (*P* = 0.327), phototype (*P* = 0.548), or duration of the disease (*P* = 0.959). 

Only the SB of those volunteers who completed the study (*n* = 40) was evaluated because the five volunteers who did not complete the study did not differ from the others. The content of the SB was analyzed, and 20.54% of the FA peaks obtained were not detected in the commercially used fatty acid standard (their amounts were under than 0.1%). C4:0, C6:0, C8:0, C10:0, C11:0, C20:3n6, and C20:4n6 FAs were not found at any time during the study in any of the groups. The average FA composition found on D0 is presented in Table 1.

Table 2 contains a summary of changes in the FAs of the study participants determined using GC-DIC. It should be noted that no statistically significant changes were observed in the squalene pattern during the study for any of the groups (Table 3).

After the descriptive analysis, a two-way ANOVA with repeated measures was performed (Tables 4(a) and 4(b)) to compare the two different factors observed in this study: groups and visits.

**Figure 2:**
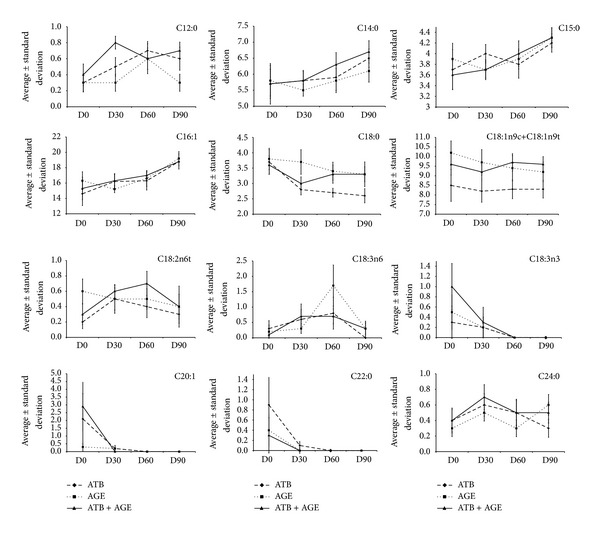
Graphic representation of fatty acids which showed differences in chromatographic pattern during the study.

Twelve of the FAs evaluated in this study showed significant changes in their contents in the SB based either on the groups and/or over the course of the visits (Figure 2). C18:1n9c+C18:1n9t showed differences only among the groups and not over the course of the visits; C14:0, C15:0, C16:1, C18:0, C18:2n6t; C18:3n6, C18:3n3; C20:1, C22:0, and C24:0 showed differences only over the course of the visits (Tables 4(a) and 4(b)). C12:0 showed differences both among the groups and over the course of the visits (Tables 4(a) and 5). This pattern was observed in all three of the groups under evaluation. During the study, C12:0 presented a higher concentration in Group 3 compared with Group 2 (*P* = 0.035), and Group 2 presented a higher concentration of C18:1n9c+C18:1n9t compared with Group 1 (*P* = 0.024) (Table 5).

Alterations in sebum patterns were observed throughout the treatments for eleven FAs in all three of the groups: C12:0 (*P* = 0.014), C14:0 (*P* = 0.031), C15:0 (*P* = 0.025), C16:1 (*P* < 0.001), C18:0 (*P* = 0.041), C18:2n6t (*P* = 0.021), C18:3n6 (*P* = 0.009), C18:3n3 (*P* = 0.011), C20:1 (*P* = 0.023), C22:0 (*P* = 0.032), and C24:0 (*P* = 0.042). 

Some FAs showed an increase in their amounts during specific visits: C12:0, from D0 to D30 and from D0 to D60 (*P* = 0.041 and *P* = 0.031, resp.); C14:0, from D30 to D90 (*P* = 0.007); C15:0, from D30 to D90 (*P* = 0.006); C16:1, from D0, D30, and D60 to D90 (*P* = 0.003, *P* < 0.001 and *P* = 0.005, resp.); C18:3n6, from D0 to D60 (*P* = 0.037); C24:0, from D0 to D30 (*P* = 0.026) (Tables 4(a) and 4(b)). However, a decrease in C18:3n6 was observed for all three of the groups from D60 to D90 (*P* = 0.042) (Tables 4(a) and 4(b)). Nevertheless, C18:0, C18:2n6t, C18:3n3, C20:1, and C22:0 had statistically significant changes on at least one visit for all three of the groups, but it was not possible to identify when the change occurred (Tables 4(a) and 4(b)).

## 4. Discussion

FAs form the lipids in SB [24–26]. On the face of an adult with regular skin, the FAs with the highest concentrations in the SB are, in decreasing order, C16:0, C18:1, C16:1+C18:2, C14:0, and C18:0. The C18:1, C18:2, and C16:1 FAs are more commonly found in the T-zone (the space including the forehead, nose, and chin), whereas C18:0 is found in the U-zone (the remainder of the face, surrounding the T-zone) [27].

In this study, neither lymecycline, nor FA supplementation were able to markedly influence FA levels in sebum. They slightly influenced the FAs levels in the patients with AV evaluated.

There are no reports in the published literature on the evaluation of the behavior of FAs in the SB of patients with acne who are being treated with an antibiotic regime based on LM. 

A previous study evaluated supplementation with the same daily dose of oral FAs as that studied herein (for the same follow-up period of 90 days) in patients with AV and compared the use of FAs to the use of a placebo [10]. Despite the absence of differences in the level of clinical improvement of acne between the groups, there was a reduction in the volume of FAs at the end of the treatment with these FA, relative to the FA volumes in the placebo group observed in a histological analyses conducted by a Dermatophatologist. These findings suggest that there are possible clinical benefits from the use of these products that can be maximized by adjusting the dosage or treatment duration [10].

On D0, the FAs that were present at higher than average concentrations in our volunteers' SB were C16:0 (17.92%), C16:1 (15.63%), squalene (13.34%), C18:1n9c+C18:1n9t (9.5%), C14:0 (5.79%), C15:0 (3.77%), C18:0 (3.74%), C17 (2.23%), C20:1 (1.78%), and C20:0 (1.62%). We noted that this SB has a composition different from that of regular skin [27] because the squalene, C15:0, C17:0, C20:1, and C20:0 were observed in the SB of these patients with AV, whereas C18:2 was not found. 

Squalene is considered the principal lipid that is increased in the SB of patients with AV, and the production of squalene is performed directly by sebocytes [28–30]. The presence of squalene is a marker of the likelihood and severity of AV because this compound enhances the comedogenicity and proliferation of *P. acnes* [24–31].

On D0, the average squalene level was 13.34%, similar to that reported in the literature (10% to 20%); [31, 32] in patients without AV, the average squalene level is 15% [31]. This result does not support the findings of Kotani and Kusu, 2002 [27], who did not find squalene in the SB of their volunteers without AV. During the treatment of the different groups, the squalene concentration did not vary significantly, either between groups or between visits. There are several possible explanations for this result: (1) the synthesis of squalene in the SGs [29] is independent of external agents; (2) this lipid is a constant marker of the likelihood of AV [31], regardless of the action of antibiotics and EFAs, similar to the results obtained with the use of hormonal antiandrogens [33] but different from the results for treatment with oral isotretinoin, during which squalene disappears from the SB; [34] or (3) the hot climate of this region, which stimulates the hyperproduction of sebum [35, 36] and, consequently, of squalene [29], may have influenced the results. This information conflicts with the theory of Ikaraocha et al., 2004 [37].

The most common profile of irritant FAs consists of saturated FAs between C8 and C12 [38–40]. In our study population, we did not observe FAs smaller than C12. Throughout our study, we observed changes in the profile of the SB for some FAs with chains larger than those with this level of irritation. 

C12:0 (lauric acid) is an FA with a medium chain length that has bactericidal activity against several infectious agents [41, 42], including *P. acnes*, *Staphylococcus aureus*, and *Streptococcus epidermidis* [43, 44], but no cytotoxicity to sebocytes [43, 45]. C12:0 is rarely found in the SB of healthy humans, with absolute rates of 1% to 2% [43, 46]. At all times during the treatment, a higher concentration of lauric acid was observed in Group 3 than in Group 2 (*P* = 0.035). Additionally, there were increases in the level of this FA in all groups between D0 and D30 (*P* = 0.041) and between D0 and D60 (*P* = 0.031).

C14:0 (myristic acid) represents 14% of the FFAs present in SB [27]. This FA has important antimicrobial against several bacteria (both Gram-positive and Gram-negative) and fungi [47]. The level of C14:0 in the SB of patients with AV included in this study was lower, perhaps because (1) it was diluted due to hyperseborrhea [8, 48], (2) it promoted a skin microenvironment prone to infection by *P. acnes* in individuals with AV, and [1–3] (3) the amount of this FA in SB decreases in age groups with higher incidences of AV [24]. We observed an increase in the myristic acid level in all groups from D30 to D90 (*P* = 0.007). This change could represent a bactericidal profile in the SB due to the use of the treatments studied in this clinical trial.

C15:0 (pentadecanoic acid) is an unusual FA found in human SB. The main source of C15:0 in humans is milk ingestion (human beings cannot synthetize C15:0 on their own), and it is stored in fatty tissues. C15:0 is an FA that is characteristically found as a constituent lipid in *P. acnes*. Our findings were different than those of Saino et al. (1976) [49], who suggested that it was possible to predict that an incremental increase in the amount of *P. acnes* could lead to worse acne lesions. Even in Groups 1 and 3 (which were treated with lymecycline), an increase in the C15:0 levels from D30 to D90 (*P* = 0.006) was observed, which could indicate that pentadecanoic acid is not an important constitutional element for this bacteria or that it is, in fact, a marker of unfeasible (devitalized) bacteria.

C16:1 (palmitoleic acid) is primarily observed in SB from the forehead and nose, and the amount of this FA decreases over time [27, 50]. C16:1 has antibacterial activity against nasal (*Pseudomonas aeruginosa*), skin (*Staphylococcus aureus*, *Streptococcus salivarius*, and *Fusobacterium nucleatum*), and oral bacteria (*Streptococcus mutans*, *Candida albicans*, *Aggregatibacter actinomycetemcomitans*, *Fusobacterium nucleatum*, and *Porphyromonas gingivalis*) but not against *P. acnes* [46, 51, 52]. The application of C16:1 to the skin of guinea pig disrupts the skin barrier, enabling the influx of Ca^2+^ into the interior of the keratinocytes, resulting in proliferation and comedogenesis [53, 54].

In healthy males, C16:1, when added by LA, represents 16.4% of the Fas in the SB [27]. In our work, the average concentration of C16:1 was 15.63% of the FAs. At the end of the study, we verified that there was an increase of the level of this FA in the SB of the volunteers of all groups between D0 and D90 (*P* = 0.003). There were also significant increases in the level of this FA between D30 and D90 (*P* < 0.001) and between D60 and D90 (*P* = 0.005).

C18:0 (stearic acid) acts to maintain the skin's pH and the integrity of the skin barrier [55]. It has no anti-inflammatory effect because it does not activate peroxisome proliferator activated receptor-*α* agonists (PPAR-*α*) [56]. FAs C18:0, C18:1, and C18:2 are closely linked to a rare skin cytosolic protein, epidermal fatty acid-binding protein (E-FABP), which is related to keratinocytic differentiation; C18:0 also participates in the synthesis of sphingolipids, which maintain the integrity of the epidermal membranes of keratinocytes and assist in the formation of ceramides [57–59]. In our volunteers, we verified that the average concentration of C18:0 was 3.74% which is lower than the 7.8% concentration found by Kotani and Kusu (2002) [27]. We observed that the concentration on at least one visit was statistically significantly different than the concentrations on the other visits in all three of the groups, but we could not identify at which visit this difference occurred. 

C18:1n9c (oleic acid) has a concentration of 17% in normal human SB [27]. This FFA has bactericidal activity against methicillin-resistant *Staphylococcus aureus* [60]. It has been hypothesized that this FA may adhere to *P. acnes* in the interior of the pilosebaceous follicles, after action of the bacterial lipase [61]. The anti-*P. acnes* activity of this FA has already been explained by the ability of this FA to increase the *β*-defensin 2 expression by sebocytes [43, 62]. In addition, this FA decreases the *in vitro *expression of keratinocytic proinflammatory cytokines (TNF-*α*, I-L-8, and IL-1) and fibroblastic cytokines (IL-8) after UVB exposure [63, 64].

At high topical concentrations, C18:1n9c alters the skin barrier [65–70], which explains why the content of this FA is increased in the type-1 skin ceramides during the winter [71]. C18:1n9c alters the skin barrier because it increases the influx of Ca^2+^ into the keratinocytes [53, 72–74]. Elaidic acid (C18:1n9t) has an important effect on the extracellular matrix because it inhibits metalloproteinase-A (MMP-A) and MMP-B, which, respectively, degrade collagen and elastic fibers. In addition, C18:1n9t activates TGF-beta (a protector of collagen) and inhibits the activation of pro-MMP-3 by plasmin (which triggers the degradation of collagen) [75–77].

The combined detection of C18:1n9c+C18:1n9t occurs because the retention times for these two compounds in the GC-DIC analysis are similar. In our study, Group 2 exhibited a higher level of association of these FAs than Group 1 (*P* = 0.024). In addition, there was a trend of improved findings when FAs were used in combination with LM (*P* = 0.051). 

C18:2n6t (LA) is an important EFA found on the skin that decreases with age (it is 40% lower in the elderly) [50]. LA is hyperconcentrated in the SB in AV, which is likely secondary to hyperseborrhea [8, 26, 78–80], leading to a scarcity of these ceramides in comedones [81]. Whenever there is a nutritional deficiency of LA and *α*-linolenic acid, these fatty acids are replaced with palmitoleic and oleic acids to form eicosatrienoic acids [82]. The proportion of LA in the SB of patients with AV is approximately 0.3% [7]; in our volunteers, however, its concentration was 0.85% when both the *cis* and *trans *forms were added together. In at least one visit, a statistically significant difference was found in the LA concentration in relation to the other visits, but it was not possible to identify when this difference occurred. Indeed, we cannot forget that SB alterations in this FA in SB are not unusual, which led us to suspect the following:

1. the exogenous administration of supplemental LM and/or LA is not capable of altering the levels of this EFA in the SB because these levels are far lower than what is observed with the use of oral isotretinoin [34] or it association with cyproterone acetate or ethynylestradiol use, [83] which are treatments to reduce sebaceous excretion and to increase the proportion of the excretion that is sebaceous;
2. the absorption of LA by the SG at the time of oral administration either does not occur or is not sustained over time. There are three potential mechanisms for this possibility: (1) 1,200 mg/day is not sufficient; (2) the LA produced in the SG is completely dependent on sebocytes for the synthesis of joined carbon fragments; or (3) for an effect to be observed, LA must be ingested for a period longer than the 90 days of this study;
3. exogenous LA has the capacity to increase the synthesis of SB by the sebocytes by stimulating PPAR-*δ*, PPAR-*α* and *stearoyl-coenzyme A desaturase* [84–86], which leads to the dilution of the LA itself as well as of other FFAs of the SB [8, 26, 48, 78–80];
4. the ingestion of high dosages of LA has an effect on the maintenance of the skin barrier, which is clinically expressed as a reduction in xerosis exclusively;
5. the ingestion of LA causes the deposition of one of its desaturation/elongation subproducts in the skin and/or other organs, which has been observed in animal experiments (joint administration of LA and *α*-linolenic acid) [87];
6. exogenously administered LA is directed to the replacement of C16:1n9 and C18:1n9 as needed to replenish them in cases of deficiency [82], thereby decreasing the availability of LA in the SB;
7. administration of oleic and *γ*-linolenic acids together with LA may interfere with their absorption by the SG.

*γ*-linolenic acid (C18:3n6) is the first product of the intracorporeal saturation of LA [18, 19, 88]. There are no data in the scientific literature regarding the level of *γ*-linolenic acid in the SB of patients with AV. In our laboratory study, this FA was found at an average percentage of 0.2%. In animals, the ingestion of *γ*-linolenic acid triggers a reduction in epidermal hyperproliferation by stimulating ceramide production [89], thus maintaining the integrity of the skin barrier [90]. Perhaps, for this reason, *γ*-linolenic acid reduces the epidermal complications of radiotherapy [91]. In humans, the ingestion of *γ*- and *α*-linoleic acids reduces skin inflammation because these FAs decrease the production of PGE-2 by blood mononuclear cells and suppress proliferation [92, 93]. For these reasons, the topical use of *γ*-linolenic acid at 2.2% soothes uremic itching [94]. In our study, for all groups, the level of C18:3n6 in the SB decreased from D0 to D60 (*P* = 0.037) and from D60 to D90 (*P* = 0.042).

C18:3n3 (*α*-linoleic acid) is rarely found in the adipose tissues of mammals due to its rapid metabolism into docosahexaenoic acid and/or beta-oxidation to acetyl-CoA and CO_2_ [95]. C18:3n3 is an EFA; thus, it must be obtained through food [5, 6, 13, 20, 21, 79, 80]. No information is available about its participation in the SB in either normal patients or in patients with AV. In our study population, the C18:3n3 concentration was 0.52%. Oral ingestion of C18:3n3 assists lymphocytic functioning [96], promotes an anti-inflammatory state [93, 97], inhibits coagulase-negative *Staphylococcus aureus* [98], and maintains the integrity of the skin barrier [90, 99, 100]. On at least one visit, a statistically significant difference was found in LA compared with the other visits, but it was not possible to identify when this difference occurred. Perhaps such a difference would occur in any treatment based on the inability of C18:3n3 to resist subsequent metabolic cycles [95].

C20:1 (gadoleic acid) is obtained through the ingestion of seeds (e.g., mustard seeds and hominy) and is likely derived from the action of elongases on oleic acid [101, 102]. Its concentration in human SB has not been described; in our study population, C20:1 was present at a low percentage (1.78%). On at least one visit, a statistically significant difference in LA was found in relation to the other visits, but it was not possible to identify when this difference occurred.

C22:0 (behenic acid) is poorly absorbed from food (only approximately 30%) and has low bioavailability; however, C22:0 is important for the formation of blood cholesterol [103]. It is deposited in brown fat [104] and also participates in the formation of ceramides [105]. The C22:0 concentration in human SB is not known, but in our study population, it was very scarce (approximately 0.5%). On at least one visit, a statistically significant difference was found in the C22:0 pattern in all three of the groups, but it was not possible to identify on which visit this difference occurred. 

C24:0 (lignoceric acid) is an FA that is present in several tissues and in the skin. It is highly linked to fatty acid transport protein 4 (FATP4) in the corneum layer [106]. For this reason, it and other long-chain FAs (8 to 24 carbons) are important for the maintenance of the skin barrier [107]. Despite the important of these Fas, C24:0 was the most abundant among them but represented only 0.39% of the total Fas [108]. Therefore, during the winter, when xerosis is more evident, its concentration decreases [109]. In our findings, the C24:0 concentration was 0.27%, less than that reported in the literature [109]. There was no final difference in the concentrations of C24:0 in the SB of all three groups, but we verified that there was an increase in its percentage from D0 to D30 (*P* = 0.026).

## 5. Conclusions

In this study, we verified that both LM and oral FA supplementation based on linoleic, *γ*-linolenic, and oleic acids may slightly influence the FAs levels in the SB of individuals with AV. We concluded that (1) LM and oral FA supplementation based on linoleic, *γ*-linolenic, and oleic acids may alter the profiles of some FA in the SB of patients; (2) the concentration of squalene in the SB did not decrease in any experimental group; (3) the concentrations of C12:0, C14:0, and C16:1 increased in all three groups during the treatment period; (4) LM and/or the ingestion of LA did not increase the level of LA in the SB; and (5) the concentration of *γ*-linolenic acid increased during the first 60 days of treatment and decreased during the following 30 days of treatment with LM and/or oral FA supplementation. All these FA measurements for the considerable time of 90 days will be an important reference for similar works in the future.

## Figures and Tables

**Table 1:** Distribution of patients with acne vulgaris according to the outcome of gas chromatography carried out on D0.

Fatty acid	*n* = 40	
	Average% (standard deviation)	Median% (min.–max.)
C12:0	0.32 (0.44)	0 (0.00–1.39)
C13:0	0.02 (0.08)	0 (0.00–0.45)
C14:0	5.79 (1.85)	5.94 (1.22–10.38)
C14:1	0.33 (0.41)	0 (0.00–1.16)
C15:0	3.77 (1.11)	3.97 (0.88–5.45)
C15:1	0.01 (0.05)	0 (0.00–0.34 )
C16:0	17.92 (5.31)	17.66 (4.83–29.25)
C16:1	15.63 (4.86)	16.73 (2.83–24.92)
C17:0	0.69 (0.57)	0.87 (0.00–1.59)
C17:1	2.23 (0.93)	2.36 (0.00–4.82)
C18:0	3.74 (1.27)	3.50 (1.77–6.62)
C18:1n9c+C18:1n9t	9.50 (2.59)	9.83 (3.33–15.30)
C18:2n6c	0.32 (0.51)	0 (0.00–1.80)
C18:2n6t	0.53 (1.26)	0 (0.00–5.45)
C18:2n6c+C18:2n6t	0.85 (1.31)	0 (0.00–5.45)
C18:3n6	0.20 (0.73)	0 (0.00–3.27)
C18:3n3	0.52 (1.29)	0 (0.00–4.44)
C20:0	1.62 (5.09)	0 (0.00–26.83)
C20:1	1.78 (4.71)	0 (0.00–22.09)
C20:2	0.12 (0.79)	0 (0.00–4.99)
C21:0	0.02 (0.10)	0 (0.00–0.66)
C20:5n3	0.12 (0.75)	0 (0.00–4.74)
C22:0	0.52 (1.47)	0 (0.00–6.35)
C22:1n9	0.14 (0.69)	0 (0.00–4.30)
C24:0	0.27 (0.47)	0 (0.00–1.42)
Squalene	13.34 (4.91)	12.88 (2.88–23.15)

Total identified	79.46 (14.42)	84.44 (40.44–100)

**Table 2:** Descriptive statistic of twelve fatty acids that presented changes during the study, based on group treatment and visits.

Fatty acid	Visit	Group 1 (*n* = 12)		Group 2 (*n* = 14)		Group 3 (*n* = 14)	
		Average (sd)	Median (min.–max.)	Average (sd)	Median (min.–max.)	Average (sd)	Median (min.–max.)
C12:0	D0	0.3 (0.4)	0.0 (0–0.9)	0.3 (0.4)	0.0 (0–1.4)	0.4 (0.5)	0.0 (0–1.2)
	D30	0.5 (0.4)	0.7 (0–0.9)	0.3 (0.4)	0.0 (0–0.9)	0.8 (0.3)	0.8 (0–1.4)
	D60	0.7 (0.4)	0.8 (0–1.1)	0.6 (0.7)	0.3 (0–2.1)	0.6 (0.5)	0.7 (0–1.7)
	D90	0.6 (0.5)	0.7 (0–1.3)	0.3 (0.4)	0.0 (0–1.0)	0.7 (0.4)	0.8 (0–1.1)

C14:0	D0	5.7 (2.2)	6.1 (1.2–9.4)	5.8 (1.8)	5.7 (3.4–10.4)	5.7 (1.7)	6.1 (1.5–7.3)
	D30	5.8 (1.0)	5.4 (4.7–7.8)	5.5 (0.7)	5.3 (4.5–6.8)	5.8 (1.2)	5.8 (3.3–7.9)
	D60	5.9 (1.4)	6.0 (2.9–7.7)	5.8 (1.4)	5.9 (2.6–8.0)	6.3 (1.4)	6.1 (4.5–10.4)
	D90	6.5 (1.0)	6.1 (5.0–8.2)	6.1 (1.3)	6.3 (2.9–8.3)	6.7 (1.3)	6.9 (4.9–8.7)

C15:0	D0	3.7 (1.3)	4.3 (0.9–5.1)	3.9 (1.1)	4.0 (1.9–5.5)	3.6 (1.0)	3.7 (1.0–4.7)
	D30	4.0 (0.6)	3.8 (3.1–5.4)	3.7 (0.6)	3.7 (2.9–5.4)	3.7 (0.7)	3.7 (1.9–4.8)
	D60	3.8 (0.9)	3.8 (2.0–4.8)	3.9 (0.7)	4.1 (2.1–4.8)	4.0 (0.9)	4.1 (2.8–6.3)
	D90	4.2 (0.6)	4.1 (3.4–5.5)	4.3 (0.7)	4.3 (2.2–5.4)	4.3 (0.7)	4.4 (2.8–5.2)

C16:1	D0	14.6 (5.3)	17.2 (2.8–18.3)	16.3 (4.4)	17.2 (8.6–24.9)	15.3 (4.5)	16.1 (3.5–21.1)
	D30	16.2 (3.2)	15.3 (12.5–21.4)	15.2 (1.7)	15.4 (12.4–17.9)	16.3 (3.1)	15.9 (8.9–20.9)
	D60	16.3 (4.2)	17.7 (8.0–21.2)	16.6 (3.9)	17.7 (7.1–21.0)	17.0 (2.5)	16.9 (12.3–22.1)
	D90	18.8 (3.4)	18.7 (13.2–25.1)	19.2 (3.4)	19.6 (12.2–25.1)	18.8 (3.4)	18.7 (13.2–25.1)

C18:0	D0	3.7 (1.4)	3.3 (1.8–6.6)	3.8 (1.3)	3.9 (2.3–6.5)	3.6 (1.1)	3.4 (2.5–6.0)
	D30	2.8 (0.6)	2.8 (1.9–4.2)	3.7 (1.5)	3.1 (2.1–8.0)	3.0 (0.8)	2.8 (2.2–5.2)
	D60	2.7 (0.5)	2.8 (1.9–3.5)	3.4 (1.1)	3.3 (1.9–5.3)	3.3 (1.1)	3.0 (2.2–5.4)
	D90	2.6 (0.8)	2.5 (1.9–5.0)	3.3 (1.5)	2.6 (1.7–6.4)	3.3 (0.9)	3.3 (2.0–5.4)

C18:1n9c+C18:1n9t	D0	8.5 (2.9)	8.8 (3.3–12.9)	10.2 (2.3)	10.7 (6.3–14.1)	9.6 (2.1)	9.8 (3.9–13.4)
	D30	8.2 (2.0)	8.0 (4.0–11.4)	9.7 (2.5)	9.2 (6.5–16.8)	9.2 (1.4)	9.0 (6.6–12.0)
	D60	8.3 (1.7)	8.0 (5.4–11.9)	9.4 (2.5)	9.6 (4.1–13.0)	9.7 (1.7)	9.7 (7.8–13.9)
	D90	8.3 (1.6)	7.9 (6.5–12.2)	9.2 (1.9)	8.9 (5.7–13.0)	9.6 (1.5)	9.5 (7.4–12.1)

C18:2n6t	D0	0.2 (0.3)	0.0 (0–0.8)	0.6 (0.6)	0.4 (0–1.8)	0.3 (0.5)	0.0 (0–1.3)
	D30	0.5 (0.5)	0.6 (0–1.3)	0.5 (0.7)	0.3 (0–2.4)	0.6 (0.3)	0.7 (0–1.1)
	D60	0.4 (0.5)	0.3 (0–1.1)	0.5 (0.5)	0.6 (0–1.4)	0.7 (0.6)	0.8 (0–2.3)
	D90	0.3 (0.4)	0.1 (0–1.2)	0.4 (1.0)	0.0 (0–3.6)	0.4 (0.6)	0.0 (0–1.9)

C18:3n6	D0	0.3 (0.9)	0.0 (0–3.3)	0.2 (0.8)	0.0 (0–3.0)	0.1 (0.4)	0.0 (0–1.7)
	D30	0.6 (1.1)	0.0 (0–3.7)	0.3 (0.6)	0.0 (0–1.8)	0.7 (1.5)	0.0 (0–5.5)
	D60	0.8 (1.8)	0.0 (0–6.2)	1.7 (2.5)	0.0 (0–8.2)	0.7 (0.9)	0.3 (0–2.3)
	D90	0.0 (0.1)	0.0 (0–0.5)	0.3 (0.9)	0.0 (0–3.1)	0.3 (0.7)	0.0 (0–2.1)

C18:3n3	D0	0.3 (1.1)	0.0 (0–3.9)	0.5 (1.3)	0.0 (0–4.5)	1.0 (1.7)	0.0 (0–4.4)
	D30	0.2 (0.7)	0.0 (0–2.6)	0.2 (0.8)	0.0 (0–3.1)	0.3 (1.1)	0.0 (0–3.9)
	D60	0.0 (0.0)	0.0 (0-0)	0.0 (0.0)	0.0 (0-0)	0.0 (0.0)	0.0 (0-0)
	D90	0.0 (0.0)	0.0 (0-0)	0.0 (0.0)	0.0 (0-0)	0.0 (0.1)	0.0 (0–0.4)

C20:1	D0	2.1 (5.6)	0.0 (0–19.4)	0.3 (1.1)	0.0 (0–4.2)	2.9 (5.9)	0.4 (0–22.1)
	D30	0.2 (0.7)	0.0 (0–2.4)	0.2 (0.8)	0.0 (0–3.1)	0.0 (0.0)	0.0 (0-0)
	D60	0.0 (0.1)	0.0 (0–0.5)	0.0 (0.0)	0.0 (0-0)	0.0 (0.0)	0.0 (0-0)
	D90	0.0 (0.0)	0.0 (0-0)	0.0 (0.0)	0.0 (0-0)	0.0 (0.0)	0.0 (0-0)

C22:0	D0	0.9 (1.9)	0.0 (0–6.4)	0.4 (1.2)	0.0 (0–4.5)	0.3 (1.3)	0.0 (0–4.8)
	D30	0.1 (0.3)	0.0 (0–0.9)	0.0 (0.0)	0.0 (0-0)	0.0 (0.2)	0.0 (0–0.6)
	D60	0.0 (0.0)	0.0 (0-0)	0.0 (0.0)	0.0 (0-0)	0.0 (0.1)	0.0 (0–0.4)
	D90	0.0 (0.0)	0.0 (0-0)	0.0 (0.0)	0.0 (0-0)	0.0 (0.0)	0.0 (0-0)

C24:0	D0	0.4 (0.5)	0.0 (0–1.3)	0.3 (0.4)	0.0 (0–1.0)	0.4 (0.6)	0.0 (0–1.4)
	D30	0.6 (0.5)	0.7 (0–1.6)	0.5 (0.4)	0.7 (0–1.0)	0.7 (0.6)	0.7 (0–1.8)
	D60	0.5 (0.6)	0.0 (0–1.4)	0.3 (0.4)	0.0 (0–1.0)	0.5 (0.6)	0.6 (0–1.6)
	D90	0.3 (0.4)	0.0 (0–0.9)	0.6 (0.5)	0.9 (0–1.2)	0.5 (0.5)	0.3 (0–1.7)

**Table 3:** Quantitative squalene variation during the study, based on group treatment and visit.

Fatty acid	Visit	Group 1 (*n* = 12)		Group 2 (*n* = 14)		Group 3 (*n* = 14)	
		Average (sd)	Median (min.–max.)	Average (sd)	Median (min.–max.)	Average (sd)	Median (min.–max.)
Squalene	D0	13.5 (6.0)	13.7 (3.0–23.2)	12.4 (4.1)	12.0 (5.3–20.3)	14.1 (4.9)	14.2 (2.9–20.9)
	D30	15.5 (3.5)	15.5 (9.9–21.1)	13.5 (4.0)	13.3 (4.5–21.9)	13.9 (3.7)	14.6 (6.9–19.0)
	D60	14.2 (4.1)	14.8 (7.6–19.6)	12.8 (2.8)	14.0 (7.9–16.1)	14.3 (4.3)	13.9 (7.3–25.0)
	D90	17.0 (3.2)	16.6 (13.0–24.1)	14.2 (3.2)	13.6 (8.1–19.1)	14.0 (6.0)	14.9 (1.4–23.0)

**Table:** (a)

Fatty acid	Factor	Degrees of freedom of numerator	Degrees of freedom of denominator	*F*-test	*P* value
C12:0	Group	2	37	3.578	**0.038**
	Visit	3	111	3.688	**0.014**
	Group ∗ Visit	6	111	1.361	0.237

C13:0	Group	2	37	0.524	0.597
	Visit	2	86	0.851	0.445
	Group ∗ Visit	5	86	1.525	0.195

C14:0	Group	2	37	0.422	0.659
	Visit	3	98	3.235	**0.031**
	Group ∗ Visit	5	98	0.358	0.885

C14:1	Group	2	37	0.155	0.857
	Visit	2	86	0.154	0.885
	Group ∗ Visit	5	86	0.745	0.582

C15:0	Group	2	37	0.033	0.967
	Visit	2	91	3.516	**0.025**
	Group ∗ Visit	5	91	0.375	0.862

C15:1	Group	2	37	1.406	0.258
	Visit	1	40	1.161	0.292
	Group ∗ Visit	2	40	1.103	0.345

C16:0	Group	2	37	2.270	0.117
	Visit	3	96	1.947	0.136
	Group ∗ Visit	5	96	0.284	0.926

C16:1	Group	2	37	0.290	0.750
	Visit	3	93	7.596	**<0.001**
	Group ∗ Visit	5	93	0.535	0.749

C17:0	Group	2	37	0.127	0.881
	Visit	3	102	1.599	0.198
	Group ∗ Visit	6	102	0.416	0.853

C17:1	Group	2	37	0.264	0.769
	Visit	3	102	0.634	0.581
	Group ∗ Visit	6	102	1.098	0.371

C18:0	Group	2	37	3.113	0.056
	Visit	3	105	2.905	**0.041**
	Group ∗ Visit	6	105	0.578	0.739

C18:1n9c+C18:1n9t	Group	2	37	4.612	**0.016**
	Visit	3	111	0.439	0.725
	Group ∗ Visit	6	111	0.284	0.944

C18:2n6c	Group	2	37	0.799	0.457
	Visit	3	111	2.004	0.118
	Group ∗ Visit	6	111	0.553	0.767

C18:2n6t	Group	2	37	0.147	0.864
	Visit	1	42	5.401	**0.021**
	Group ∗ Visit	2	42	0.328	0.749

C18:2n6c+C18:2n6t	Group	2	37	0.197	0.822
	Visit	2	70	2.229	0.118
	Group ∗ Visit	4	70	0.569	0.677

**Table:** (b)

Fatty acid	Factor	Degrees of freedom of numerator	Degrees of freedom of denominator	*F*-test	*P* value
C18:3n6	Group	2	37	0.377	0.689
	Visit	2	76	4.945	**0.009**
	Group ∗ Visit	4	76	0.985	0.423

C18:3n3	Group	2	37	0.626	0.541
	Visit	2	67	5.118	**0.011**
	Group ∗ Visit	4	67	0.634	0.625

C20:0	Group	2	37	0.178	0.838
	Visit	2	68	2.121	0.132
	Group ∗ Visit	4	68	0.159	0.949

C20:1	Group	2	37	0.923	0.406
	Visit	1	40	5.392	**0.023**
	Group ∗ Visit	2	40	1.099	0.347

C20:2	Group	2	37	0.925	0.406
	Visit	1	39	0.899	0.354
	Group ∗ Visit	2	39	0.925	0.410

C21:0	Group	2	37	1.299	0.285
	Visit	2	67	0.334	0.697
	Group ∗ Visit	4	67	0.402	0.789

C20:3n3	Group	2	37	0.029	0.971
	Visit	1	43	2.905	0.090
	Group ∗ Visit	2	43	0.1	0.929

C20:5n3	Group	2	37	2.133	0.133
	Visit	1	41	2.168	0.147
	Group ∗ Visit	2	41	2.550	0.085

C22:0	Group	2	37	0.535	0.590
	Visit	1	40	4.796	**0.032**
	Group ∗ Visit	2	40	0.431	0.653

C22:1n9	Group	2	37	1.177	0.319
	Visit	2	79	0.322	0.739
	Group ∗ Visit	4	79	1.145	0.343

C22:2	Group	2	37	1.002	0.377
	Visit	1	39	3.802	0.056
	Group ∗ Visit	2	39	1.002	0.380

C23:0	Group	2	37	1.177	0.319
	Visit	1	39	1.239	0.275
	Group ∗ Visit	2	39	1.177	0.321

C24:0	Group	2	37	0.254	0.777
	Visit	3	111	2.821	**0.042**
	Group ∗ Visit	6	111	1.595	0.155

Squalene	Group	2	37	1.252	0.298
	Visit	3	99	1.761	0.165
	Group ∗ Visit	5	99	0.704	0.631

Total identified	Group	2	37	1.548	0.226
	Visit	3	101	1.087	0.354
	Group ∗ Visit	5	101	0.241	0.953

**Table 5:** Multiple comparison method for C12:0 and C18:1n9c+C18:1n9t based on treatments.

Fatty acid	Comparison	Differences (estimated average)	Standard deviation	*P* value	95% Confidence interval
C12:0	Group 1 versus Group 2	0.174	0.109	0.361	(−0.100; 0.447)
	Group 1 versus Group 3	−0.105	0.109	>0.999	(−0.378; 0.169)
	Group 2 versus Group 3	−0.278	0.105	**0.035**	**(**−**0.541; **−**0.015)**

C18:1n9c+C18:1n9t	Group 1 versus Group 2	−1.341	0.479	**0.024**	**(**−**2.543; **−**0.139)**
	Group 1 versus Group 3	−1.200	0.479	0.051	(−2.402; 0.002)
	Group 2 versus Group 3	0.141	0.461	>0.999	(−1.014; 1.296)
